# Efficient numerical approaches with accelerated graphics processing unit (GPU) computations for Poisson problems and Cahn-Hilliard equations

**DOI:** 10.3934/math.20241334

**Published:** 2024-09-23

**Authors:** Saulo Orizaga, Maurice Fabien, Michael Millard

**Affiliations:** 1 Department of Mathematics, New Mexico Tech, 801 Leroy Place, Socorro, NM 87801, USA; 2 Department of Mathematics, University of Wisconsin-Madison,Van Vleck Hall, 213, 480 Lincoln Dr, Madison, WI 53706, USA

**Keywords:** phase-field models, Cahn-Hilliard equation, thin-film equation, efficient numerical methods, GPU computation, 65M99, 65T50

## Abstract

In this computational paper, we focused on the efficient numerical implementation of semi-implicit methods for models in materials science. In particular, we were interested in a class of nonlinear higher-order parabolic partial differential equations. The Cahn-Hilliard (CH) equation was chosen as a benchmark problem for our proposed methods. We first considered the Cahn-Hilliard equation with a convexity-splitting (CS) approach coupled with a backward Euler approximation of the time derivative and tested the performance against the bi-harmonic-modified (BHM) approach in terms of accuracy, order of convergence, and computation time. Higher-order time-stepping techniques that allow for the methods to increase their accuracy and order of convergence were then introduced. The proposed schemes in this paper were found to be very efficient for 2D computations. Computed dynamics in 2D and 3D are presented to demonstrate the energy-decreasing property and overall performance of the methods for longer simulation runs with a variety of initial conditions. In addition, we also present a simple yet powerful way to accelerate the computations by using MATLAB built-in commands to perform GPU implementations of the schemes. We show that it is possible to accelerate computations for the CH equation in 3D by a factor of 80, provided the hardware is capable enough.

## 1. Introduction

Phase field models are a very important class of nonlinear partial differential equations that are used to model a number of physical processes ranging from bio-medical applications to metallurgy. That was the original application considered when the Cahn-Hilliard (CH) equation was first introduced in 1958 [1]. The Cahn-Hilliard equation is a fourth-order nonlinear partial differential equation (PDE) for which explicit methods are not practical since they would require very small steps for numerical stability ($h\leq 𝒪dx^{4}$), where h is the time step and dx is the grid spacing in one dimension. On the other hand, fully implicit methods would ensure stability but can be computationally expensive for problems in more than one dimension. For this reason, a number of research works have been devoted to the efficient implementation and computation of the CH equation via semi-implicit methods. David Eyre proposed a very elegant and simple approach to compute the CH equation in what is known as the convexity-splitting (CS) approach [2]. The main idea of the method was to re-express the energy in terms of convex and concave parts, leading to energy-stable solutions for the problem. Since then, a number of extensions based on the CS approach have been implemented to solve the CH with improved order of accuracy [3–6].

Another formulation that was proposed to solve the CH equation was presented by Andrea Bertozzi [7]. The main idea is to re-express the CH equation with a bi-harmonic term that is to be computed at the implicit level. This, in turn provides another efficient computational approach termed the bi-harmonic-modified method (BHM). The BHM approach can also be understood as a splitting method with an stabilizer parameter. Another more recent formulation for the CH equation is found in the scalar auxiliary variable (SAV) approach, which requires a reformulation of the energy and the use of stabilizer parameters to produce energy-stable results [8–10].

The CH equation is an important model for various physical processes in science and engineering since it can be easily extended to the diblock copolymer (BCP) equation [11]. The BCP equation has been used to understand self-assembly properties of block copolymers and their relevance to higher-quality material production. Another extension of the CH equation is the phase-field crystal PFC equation [12], which is used to study the crystal formation at the atomic level in a solidification process. Several authors have worked in the efficient computation of the BCP and PFC equations [6, 11, 13–16]. In addition, other important applications of the CH equation are image inpainting [17] and bio-medical applications, such as, tumor growth and drug-delivery [18, 19].

The purpose of this paper is to provide a numerical approach for materials science models based on the BHM formulation that is efficient, accurate, and easy to implement for solving phase-field models. In terms of ease of implementation, we believe the CS and BHM method are the best candidates so that our methodology can be used among a broad range of researchers from different disciplines. Also, we aim to propose a simple approach to speed up computations of the proposed schemes via a graphics processing unit (GPU) implementation.

In this paper, we first compare the bi-harmonic-modified (BHM) approach against the more well-known CS approach in terms of accuracy and CPU computation time. We then construct our schemes using the BHM method and couple it, for increased accuracy, with an implicit-explict (IMEX) time stepping formulation. In Section 2, we introduce the CH equation. In Section 3, we introduce the CS, BHM, and IMEX approaches for solving the CH equation. In Section 4, we perform numerical experiments in 2D to test the performance of the algorithms and to display numerical solutions over time. In Section 5, we show how the algorithms can be adapted to 3D simulations and test their performance. Finally, we also show how to speed up 3D simulations by performing graphics processing unit (GPU) computations by using simple MATLAB commands. The main goal of this computational paper is to contribute to the acceleration of methods for related Poisson problems and CH equations in 2D and 3D.

## 2. Mathematical formulation

The Cahn-Hilliard energy is given by [1]

(2.1) $$ℰ(u)=\int_{\Omega }\frac{\epsilon^{2}}{2}|\nabla u{|}^{2}+W\left(u\right)dx,$$

where u(x,t) is the phase-field variable, which represents a volume fraction of one component, and W(u) is a double-well potential given by

(2.2) $$W\left(u\right)=\frac{1}{4}{\left(u^{2}-1\right)}^{2},$$

which gives that $W^{\prime }(u)=u^{3}-u.W(u)$ has two minima at u=1 and u=−1, which are associated with the pure state of the materials in the binary mixture; ϵ defines the transition layer thickness separating the two materials. Ω will be considered as a box, $\Omega =[0,L{]}^{d}$, in 2D or 3D (d=2,3).

The CH equation is the $H^{-1}$ gradient flow of the energy ℰ(u) that is given in terms of the chemical potential $\mu =-\epsilon^{2}\Delta u+W^{\prime }(u)$. The CH equation reads

(2.3) $$\frac{\partial u}{\partial t}=-\epsilon^{2}\Delta^{2}u+\Delta \left(u^{3}-u\right).$$

The solution u for the CH equation, at the continuous level, will evolve into configurations such that the energy is always decreasing, that is

$$\frac{d}{dt}ℰ(u)=-\int_{\Omega }|\nabla \mu {|}^{2}\leq 0.$$

For this reason, it is very important to design numerical methods that retain this property.

## 3. Numerical methods

### 3.1. Convexity-splitting method

An elegant and very efficient way to generate numerical solutions for the CH equation was proposed by Eyre in 1998 [2]. The method consists of splitting the CH energy into convex and concave parts. The energy can be split in the following way

(3.1) $$ℰ\left(u\right)=\underset{\Omega }{\int }\frac{\epsilon^{2}}{2}{\left|\nabla u\right|}^{2}+W\left(u\right)dx=\underset{\Omega }{\int }\frac{\epsilon^{2}}{2}{\left|\nabla u\right|}^{2}+\frac{au^{2}}{2}+\left(\frac{u^{4}}{4}-\left(1+a\right)\frac{u^{2}}{2}\right)dx,$$

which gives the form of the energy $ℰ(u)={ℰ}_{+}(u)+{ℰ}_{-}(u)$ in terms of a convex and concave part, provided that the splitting parameter a>2. The convex part is to be computed implicitly while the concave part is to be computed explicitly. The convexity-splitting applied to the CH equation gives

(3.2) $$\frac{\partial u}{\partial t}=\left(-\epsilon^{2}\Delta^{2}+a\Delta \right)u+\Delta \left[u^{3}-(1+a)u\right].$$

We denote time-discretized approximation of the solution as $u\left(x_{i},t_{n}\right)\approx U_{n,i}$ where the discrete times will be expressed with respect to the local timestep, h, by $t_{n+1}=t_{n}+h$. Using the backward Euler difference for the time derivative, it gives

(3.3) $$\frac{U_{n+1}-U_{n}}{h}=\left(-\epsilon^{2}\Delta^{2}+a\Delta \right)U_{n+1}+\Delta \left[U_{n}^{3}-(1+a)U_{n}\right],$$

which is called the convexity-splitting CS scheme. For clarity of presentation, we show the form of the CS scheme in Fourier pseudo-spectral notation [20]. The approximation for U on [0,2π] takes the form (2D case)

$$U\approx \sum_{k_{x}=1}^{N}\sum_{k_{y}=1}^{N}\hat{U}\left(k_{x},k_{y},t\right)\text{exp}\left[2\pi i\left(\omega \left(k_{x}\right)x+\omega \left(k_{y}\right)y\right)\right],$$

where $\omega \left(k_{x}\right),\omega \left(k_{y}\right)$ are the wave-numbers, $k^{2}=\omega {\left(k_{x}\right)}^{2}+\omega {\left(k_{y}\right)}^{2}$, and $\hat{U}$ is computed using the discrete Fourier transform. Applying the Fourier transform to (3.3)

(3.4) $$\frac{{\hat{U}}_{n+1}-{\hat{U}}_{n}}{h}=\left(-\epsilon^{2}k^{4}-ak^{2}\right){\hat{U}}_{n+1}-k^{2}\left[\hat{U_{n}^{3}}-\left(1+a\right){\hat{U}}_{n}\right],$$

Using $\hat{\Delta^{2}u}=k^{4}\hat{u}$, $\hat{\Delta u}=-k^{2}\hat{u}$, and solving for ${\hat{U}}_{n+1}$, one gets

(3.5) $${\hat{U}}_{n+1}=\frac{{\hat{U}}_{n}-hk^{2}\left[\hat{U_{n}^{3}}-(1+a){\hat{U}}_{n}\right]}{1+h\left(\epsilon^{2}k^{4}+ak^{2}\right)},$$

where the inverse Fourier transform is applied to the above scheme to obtain $U_{n+1}$. For more details in the CS method and for an energy decreasing proof, the readers are referred to [6, 21].

### 3.2. Bi-harmonic-modified (BHM) method

Another important splitting leading to energy-decreasing property was proposed by Bertozzi for CH equations with variable mobility [7]. We start with the CH equation written as

(3.6) $$\frac{\partial u}{\partial t}=\nabla \cdot \left(M\left(u\right)\nabla \left[-\epsilon^{2}\Delta u+W^{\prime }\left(u\right)\right]\right),$$

where M(u) is the variable mobility. We then distribute the divergence operator

(3.7) $$\frac{\partial u}{\partial t}=\nabla \cdot \left(M\left(u\right)\nabla \left[-\epsilon^{2}\Delta u\right]\right)+\nabla \cdot \left(M\left(u\right)\nabla W^{\prime }\left(u\right)\right),$$

and introduce a splitting parameter $M_{1}$ to re-express the first mobility function that appears in the above equation with $M(u)=M(u)-M_{1}+M_{1}$, which gives

(3.8) $$\frac{\partial u}{\partial t}=-M_{1}\epsilon^{2}\Delta^{2}u+\epsilon^{2}\nabla \cdot \left[\left(M\left(u\right)-M_{1}\right)\nabla \Delta u\right]+\nabla \cdot \left[M\left(u\right)\nabla W^{\prime }\left(u\right)\right],$$

which gives the original BHM approach applied to the CH equation with variable mobility. However, if we consider the case where M(u) is a constant, then the splitting reduces to the following

(3.9) $$\frac{\partial u}{\partial t}=-M_{1}\epsilon^{2}\Delta^{2}u-\epsilon^{2}\left(M\left(u\right)-M_{1}\right)\Delta^{2}u+M\left(u\right)\Delta W^{\prime }\left(u\right),$$

where the bi-harmonic term is to be computed implicitly. We then approximate the time derivative to arrive at the BHM scheme, which reads

(3.10) $$\frac{U_{n+1}-U_{n}}{h}=-M_{1}\epsilon^{2}\Delta^{2}U_{n+1}-\epsilon^{2}\left(M\left(u\right)-M_{1}\right)\Delta^{2}U_{n}+M\left(u\right)\Delta \left(U_{n}^{3}-U_{n}\right),$$

where we will always assume, for the rest of the paper, that M(u)=1. We note that (3.10) is a scheme that, to the best of our knowledge, has not been applied to the CH equation with constant mobility, since this case of CH equation has traditionally been computed using the celebrated CS method. We also remark that the BHM method was originally formulated for the case of variable mobility [7]. The Fourier pseudo-spectral notation for the BHM scheme in (3.10) then reads

(3.11) $$\hat{U_{n+1}}=\frac{\hat{U_{n}}-h\epsilon^{2}\left(M(u)-M_{1}\right)k^{2}\hat{U_{n}}-M(u)k^{2}\left[\hat{U_{n}^{3}}-\hat{U_{n}}\right]}{1+hM_{1}\epsilon^{2}k^{4}}.$$

For more details on the BHM method and related applications of phase-field models to fluid dynamics, the readers are referred to the work of Bertozzi [7].

### 3.3. Stability of the schemes: BHM and CS

For a stability criteria of the BHM method given by (3.11), we will use the approach presented in [22]. Equation (3.9) is of the form $u_{t}=G(u)$, where G(u)=Ψ(u)+Φ(u). Here, $\Psi (u)=-M_{1}\epsilon^{2}\Delta^{2}u$ and $\Phi (u)=-\epsilon^{2}\left(M(u)-M_{1}\right)\Delta^{2}u+M(u)\Delta W^{\prime }(u)$. Using the expression $\hat{\Phi \left(U_{n}\right)}=\hat{G\left(U_{n}\right)}-\hat{\Psi \left(U_{n}\right)}$ in (3.11) gives the following

(3.12) $$\hat{U_{n+1}}=\hat{U_{n}}+\frac{h\hat{G\left(U_{n}\right)}}{1+hM_{1}\epsilon^{2}k^{4}}.$$

To analyze the conditions for linear stability, we consider the highest order terms in G(u) to arrive at the approximation $\hat{G\left(U_{n}+e_{n}\right)}\approx \hat{G\left(U_{n}\right)}-C_{o}\epsilon^{2}k^{4}{\hat{e}}_{n}$, which is then used in (3.12) to obtain the amplification factor associated with the growth of the errors in $U_{n}$. The amplification factor is given by

(3.13) $$\sigma =1-\frac{hC_{o}\epsilon^{2}k^{4}}{1+hM_{1}\epsilon^{2}k^{4}}.$$

The conditions to guarantee stability of the THM scheme, require that |σ|<1, so one gets

$$-1<1-\frac{hC_{o}\epsilon^{2}k^{4}}{1+hM_{1}\epsilon^{2}k^{4}}<1,-2<-\frac{hC_{o}\epsilon^{2}k^{4}}{1+hM_{1}\epsilon^{2}k^{4}}<0.$$

Given the quantities considered in this formulation, the less than zero inequality is satisfied, so one works with

$$-2\left(1+hM_{1}\epsilon^{2}k^{4}\right)<-hC_{o}\epsilon^{2}k^{4},$$

which can be further reduced by re-arranging the terms to arrive at the following inequality

$$\epsilon^{2}hk^{4}\left(C_{o}-2M_{1}\right)<2.$$

In order to guarantee stability (|σ|<1), we satisfy the above inequality with the requirement that $\left(C_{o}-2M_{1}\right)<0$, which gives $M_{1}>C_{o}/2$. In the context of a constant mobility case, we can set $C_{o}=M$, which gives a minimum value for the splitting parameter in the BHM method (3.11). We note that in the analysis performed in [7] a criteria of $M_{1}>M$ was obtained for the original BHM approach. Following the approach in [22], we are able to get an improved criteria for this splitting parameter threshold. We will use $M_{1}>M/2$ for all the simulations presented in this paper. Similarly, for the CS method, we are able to obtain a stability criteria for the CS parameter a>2 to guarantee energy decreasing property for the scheme. With regards to further details on the CS method and the corresponding energy decreasing property of the CS method, an elegant proof can be found in the work presented by Shen [21]. In addition, a proof based on a functional formulation can be found in [6]. Furthermore, for the two mentioned splitting approaches, it has been shown computationally that reducing the values of the splitting parameters tends to improve accuracy at the expense of introducing numerical instability, while increasing the splitting parameter increases error and provides numerical stability [6, 23].

### 3.4. IMEX methods

IMEX methods can be understood as time-stepping techniques that are useful after a splitting has taken place in a given model equation. IMEX schemes are known to preserve the energy decreasing property of original formulations (CS and BHM splittings) while increasing the temporal accuracy [23, 24]. Our schemes given by (3.3) and (3.10) can be represented in the following form

(3.14) $$u_{t}=\Psi \left(U_{n+1}\right)+\Phi \left(U_{n}\right),$$

which are then suitable for an implicit-explicit (IMEX) Runge-Kutta (RK) time-stepping technique [25, 26]. The main idea of the IMEX formulation is to treat Ψ implicitly and the stiff term Φ explicitly. The IMEX schemes read

(3.15) $$U^{\left(1\right)}\;=U_{n}+h\left(\alpha \Psi \left(U^{\left(1\right)}\right)+\alpha \Phi \left(U_{n}\right)\right),$$

(3.16) $$U_{n+1}\;=U_{n}+h\left(\alpha \Psi \left(U_{\left(n+1\right)}+\beta \Psi \left(U^{\left(1\right)}\right)+\gamma \Phi \left(U^{\left(1\right)}\right)+\omega \Phi \left(U_{\left(n\right)}\right)\right)\right.,$$

where $\alpha =(2-\sqrt{2})/2$, $\beta =\sqrt{2}/2$, $\gamma =1/(2-\sqrt{2})$ and $\omega =(1-\sqrt{2})/(2-\sqrt{2})$. This scheme is a stage 2 scheme and is of second-order accuracy. Several authors have implemented IMEX methods for solving phase-field models in the past. Song [5] proposed an IMEX time-stepping approach for the CH equation. Ceniceros [27] proposed an IMEX time-stepping approach for a CH equation with variable mobility. The IMEX schemes for the BHM approach are different than all the mentioned ones since those authors did not rely on the BHM approach as a basis for their splitting.

## 4. Numerical experiments I

The CS method was originally developed for the CH equation with constant mobility, M(u)=1, and the method, which is an order-one scheme, has seen a number of improvements in terms of accuracy. For example, the authors in [6] proposed several schemes that improved the accuracy of the CS method with the use of several extrapolation techniques, iterations, and different time stepping techniques based on backward differentiation formulas (BDF) [28]. In this section, we will perform a direct comparison between the basic form of the CS and BHM method in terms of accuracy. All numerical experiments will be performed on a box subject to periodic boundary conditions, $\Omega =[0,2\pi {]}^{d}$, d=2,3 for 2D or 3D space dimensions.

### 4.1. CS and BHM errors

**Figure 1.**
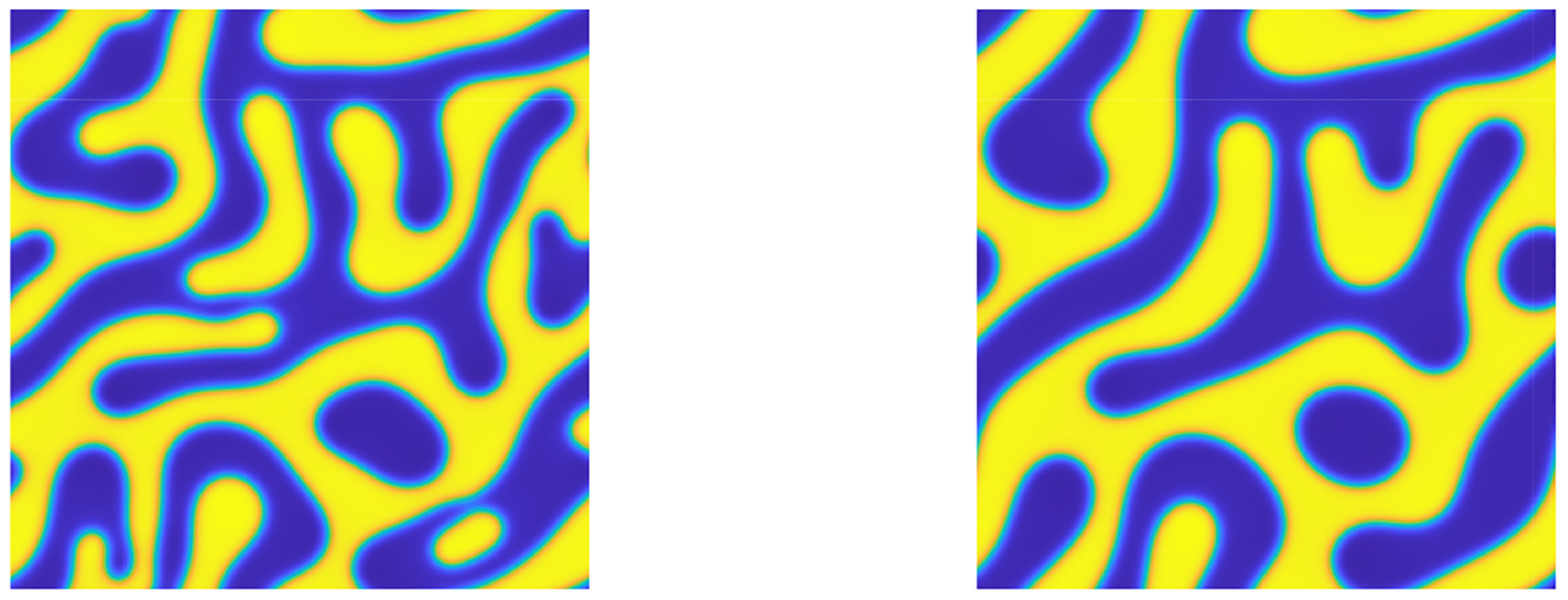
Numerical solution to the CH equation in 2D with random initial conditions using BHM method with parameters ϵ=0.05, N=256, h=0.01M=1, $M_{1}=5$, $\Omega =[0,2\pi {]}^{2}$. Left figure at t=0 and right figure at $t_{f}=1.0$.

We compare the performance of the CS and BHM scheme by solving the CH equation (2.3) with d=2 on $\Omega =[0,2\pi {]}^{2}$ subject to periodic boundary conditions using the initial condition

(4.1) $$u\left(x,y,0\right)=0.1+0.01\text{rand}\left(x,y\right),$$

which is a suitable benchmark problem for capturing the phase separation of two materials. The random initial condition was evolved for one second to exhibit a well-defined separation process and this solution state was used as the actual initial condition. The simulation using the BHM scheme (3.10) with ϵ=0.05, M=1, $M_{1}=5$, and $t_{f}=1.0$ is presented in Figure 1. The solution of the pure state u≈1 is represented in yellow and u≈−1 is represented in blue.

An accurate reference solution $U_{ref}(x,y,t)$ was constructed by using the BHM scheme with a small timestep size of $h=10^{-6}$ and a final configuration of $t_{f}=1.0$. We then compute the numerical solution using CS and BHM scheme with difference choices of h and compare against the reference solution at $t_{f}=1.0$. Computing the error with the formula:

(4.2) $$\text{Error}\left(h\right)=\int_{\Omega }\left|U\left(x,y,t_{f};h\right)-U_{ref}\left(x,y,t_{f}\right)\right|dx.$$

**Figure 2.**
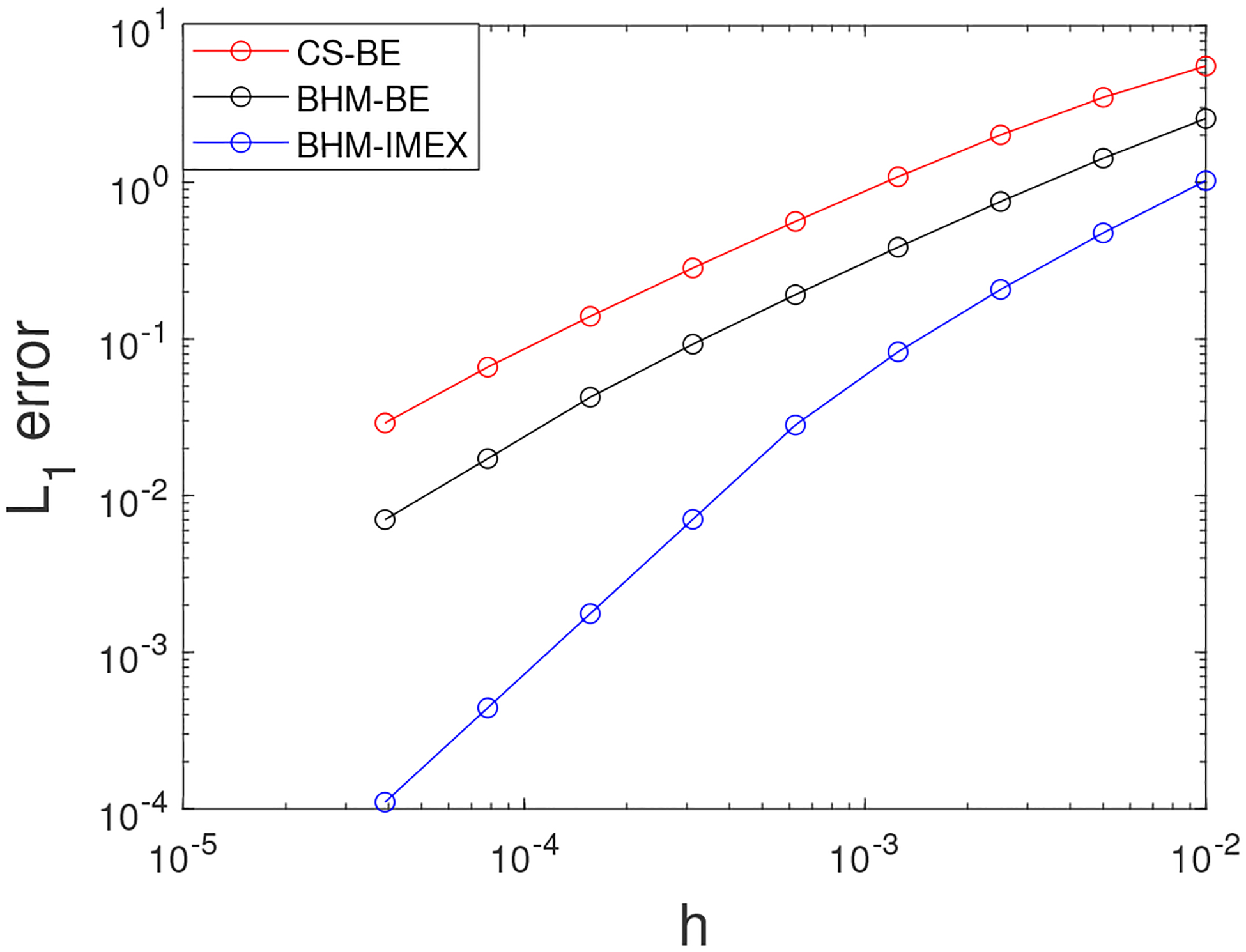
Computed errors for the CH equation in 2D using CS and BHM method and BHM-IMEX with parameters ϵ=0.05, N=256, M=1, $M_{1}=5$, $\Omega =[0,2\pi {]}^{2}$.

The computed errors are presented in Figure 2. The error plot demonstrates that both schemes are able to produce errors that follow the expected order-one convergence, and the errors produced by the BHM scheme are found to be smaller than those obtained by the CS scheme. In summary, we found that the BHM is not only reliable to solve the CH with constant mobility but is also an improvement over the CS method. The BHM-IMEX scheme is used to showcase the ability for the BHM scheme to achieve smaller errors while reaching second-order accuracy (see blue error plot in Figure 2).

### 4.2. CS and BHM computation time

**Figure 3.**
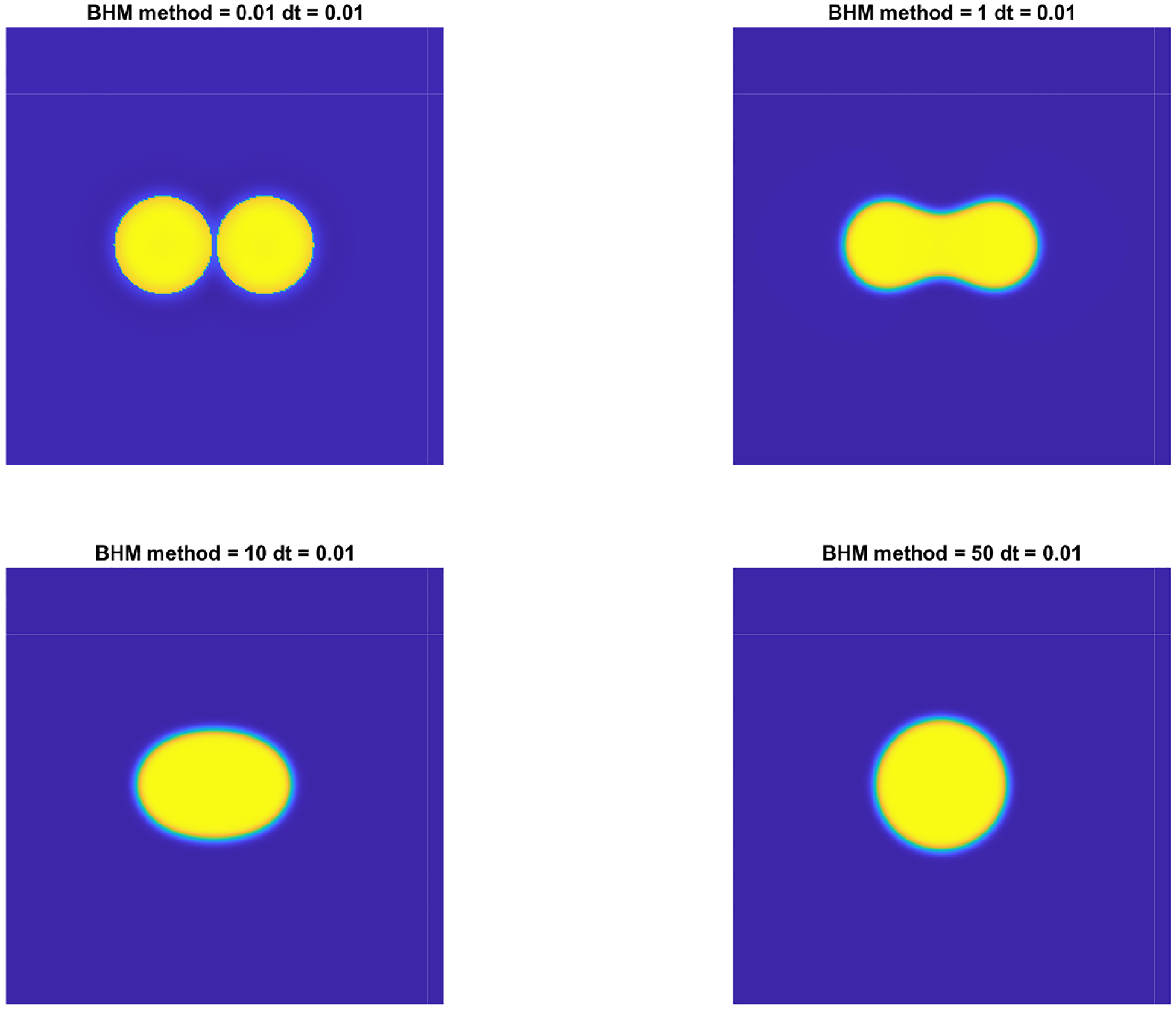
Numerical solution to the CH equation (2.3) in 2D illustrating the collision of two drops using the BHM method with parameters ϵ=0.05, N=256, h=0.01M=1, $M_{1}=5$, $\Omega =[0,2\pi {]}^{2}$.

**Figure 4.**
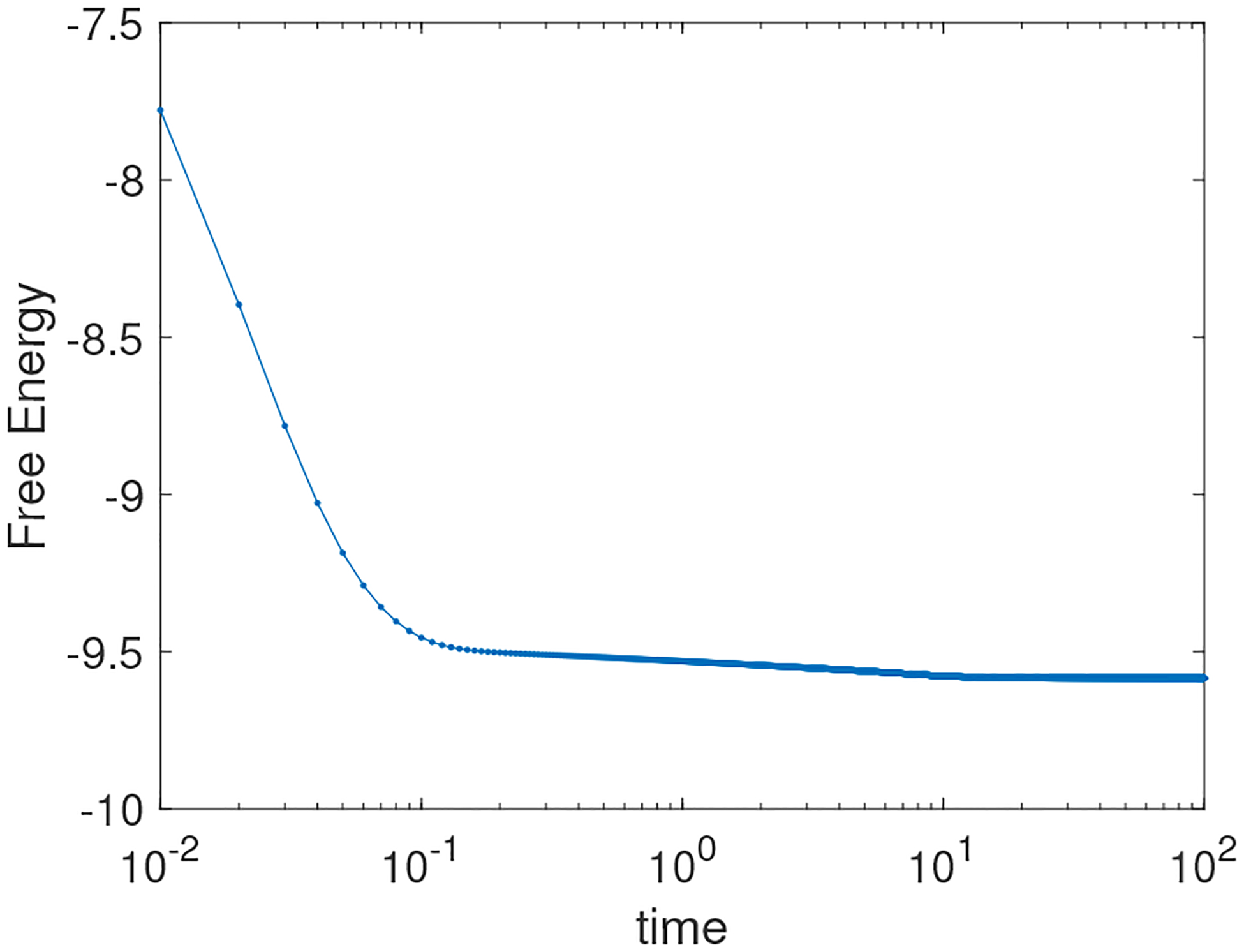
Energy evolution corresponding to the simulation presented in Figure 3.

We also include another benchmark problem for the CH equation (2.3) in 2D by simulating the coalescence of two drops in $\Omega =[0,2\pi {]}^{2}$. The initial condition is given by

(4.3) $$u\left(x,y,t=0\right)=\{\begin{array}{ll} 1, & {\left(L/9\right)}^{2}<{\left(x-L/2.8\right)}^{2}+{\left(y-L/2\right)}^{2},\\ 1, & {\left(L/9\right)}^{2}<{\left(x-b/1.7\right)}^{2}+{\left(y-L/2\right)}^{2},\\ -1, & \text{otherwise,} \end{array}$$

where L=2π. The simulation results using the BHM approach are presented in Figure 3. The corresponding energy evolution is presented in Figure 4. The merging of the drops starts around t=1. Then, by the action of mass combination, the drops exhibit an elliptical shape around t=10, which is then followed by formation of a circular drop around time t=20. We test for the computation time for the CS and BHM methods to solve the drop collision problem with a final time of $t_{f}=50$. Results are presented in Table 1. The results indicate that the BHM method can be slightly more computationally expensive than the CS method, but this comes with benefits of added accuracy. The IMEX formulation is not much more expensive than BHM for the case of N=1024, which allows for errors to reach second-order accuracy. We also note that the methods presented in this paper, namely CS, BHM, and BHM-IMEX (coupled with a backward Euler approximation), are semi-implicit methods, which are very efficient for 2D problems and can run on a modest laptop [6, 23].

## 5. Numerical experiments II

Now that the schemes have been shown to produce accurate results in 2D, we demonstrate the capability of the schemes for working in full 3D space variables. In addition, this section also aims to present the efficient computation and simulation of our schemes via GPU implementation.

### 5.1. Poisson problem

The first experiment we present for timing of computations is the Poisson problem in 2D subject to periodic boundary conditions. This numerical experiment is relevant since the Poisson problem is often encountered as one solves more complicated materials science models, very useful as a benchmark problem [13].


(5.1) $$\Delta u=f\left(x,y\right).$$

For the above problem, we follow the method of manufactured solutions [29–31]. The problem is solved on $\Omega =[0,2\pi {]}^{2}$. The Fourier spectral scheme [20] for Eq (5.1) is then formulated as

(5.2) $$\hat{\Delta u}=\hat{f}.$$

Using $\hat{\Delta u}=-k^{2}\hat{u}$, where k represents the vector containing the wave numbers for the x and y direction, the following equation is given

(5.3) $$k^{2}\hat{u}=-\hat{f}.$$

**Figure 5.**
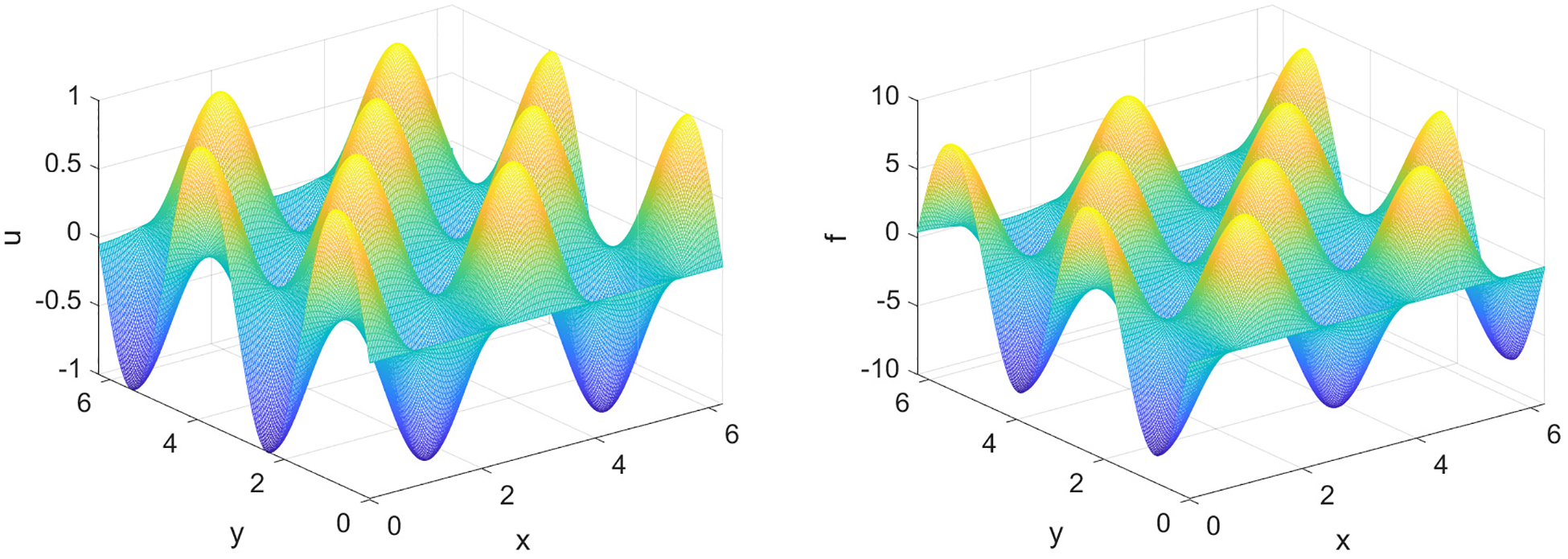
Surface plots for u(x,y) and f(x,y) corresponding to Eq (5.1) with N=256.

The equation given by (5.3) can be solved as $\hat{u}=-\hat{f}/k^{2}$; then, use the inverse fast Fourier transform (IFFT) to obtain the solution u for (5.1). We note, however, that the zero Fourier mode will generate an issue during the inversion process. It is possible to simply consider the minimization of the quantity (Δu−f) since f is provided and u was manufactured; we can approximate $\Delta u\approx \text{IFFT}\left(-k^{2}\hat{u}\right)$. Another option that would allow to complete the inversion step in the scheme is to solve a modified or perturbed Poisson problem

(5.4) $$\Delta u+\epsilon u=f\left(x,y\right),$$

where ϵ=0.1, the exact solution is u=cos⁡(2x)sin⁡(2y) and $f\left(x,y\right)=-7.9\text{cos}(2x)\text{sin}(2y)$. The corresponding formula for $\hat{u}$ is given by

(5.5) $$\hat{u}=\frac{\hat{f}}{-k^{2}+\epsilon },$$

which is now able to be fully inverted via IFFT. The surface plots for u and f are presented in Figure 5 and the computation times for various values of N are given in Table 2.

**Figure 6.**
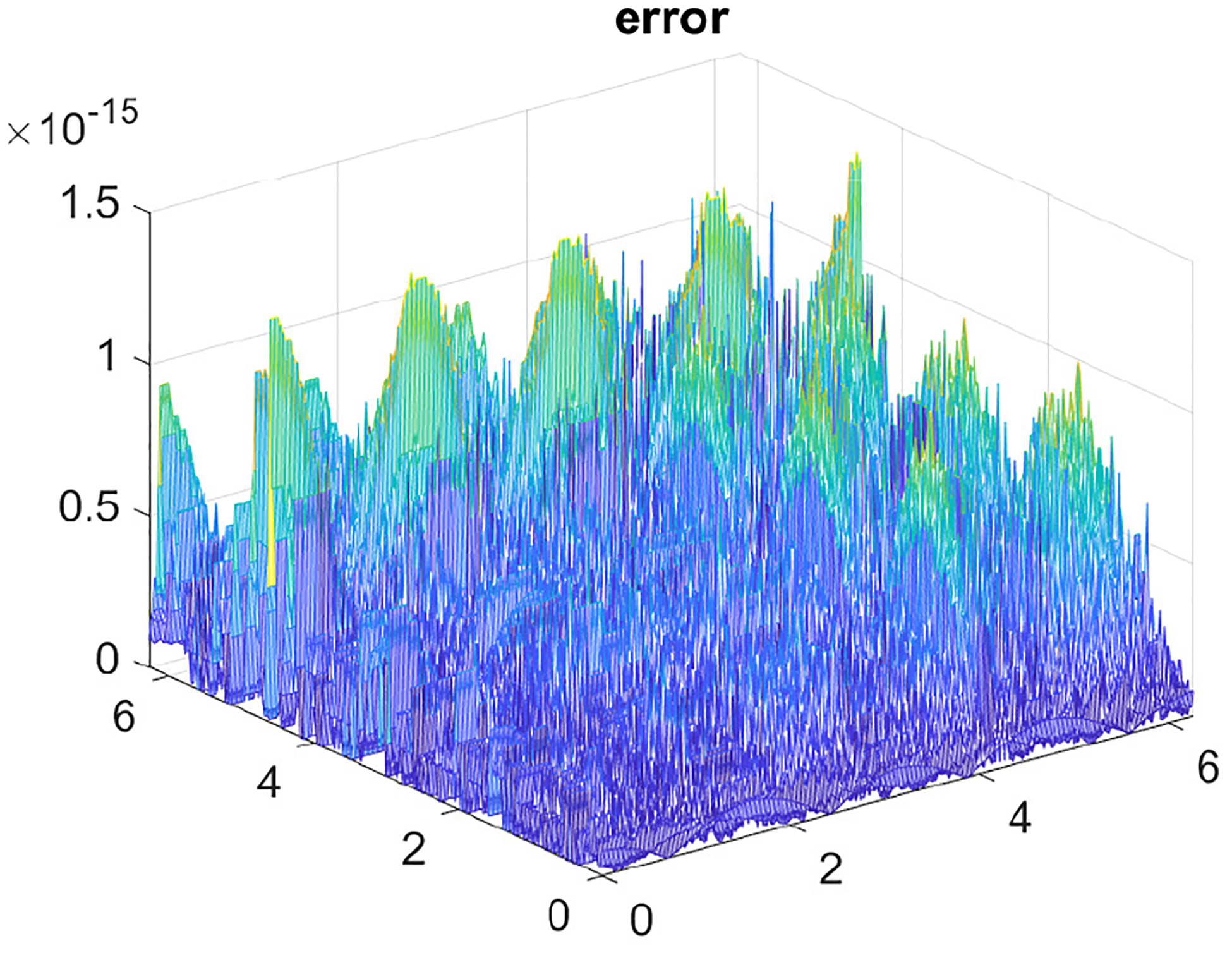
Surface error plot corresponding to Eq (5.1).

The computations for the 2D Poisson problem are very efficient and our CPU is able to handle high resolution grids, N=8192, in just under 2 seconds. This is a considerable task since for this value of N, our 2D array has $N^{2}$ entries. The efficiency in computation is attributed to the fast Fourier transform (FFT), which is highly optimized in MATLAB. Regarding the spectral error for our test problem, as we varied N for larger sizes, decreasing h, the errors remained under a 10^−15^ threshold. A representative error plot is given in Figure 6 with N=256.

### 5.2. Poisson problem in 3D

**Figure 7.**
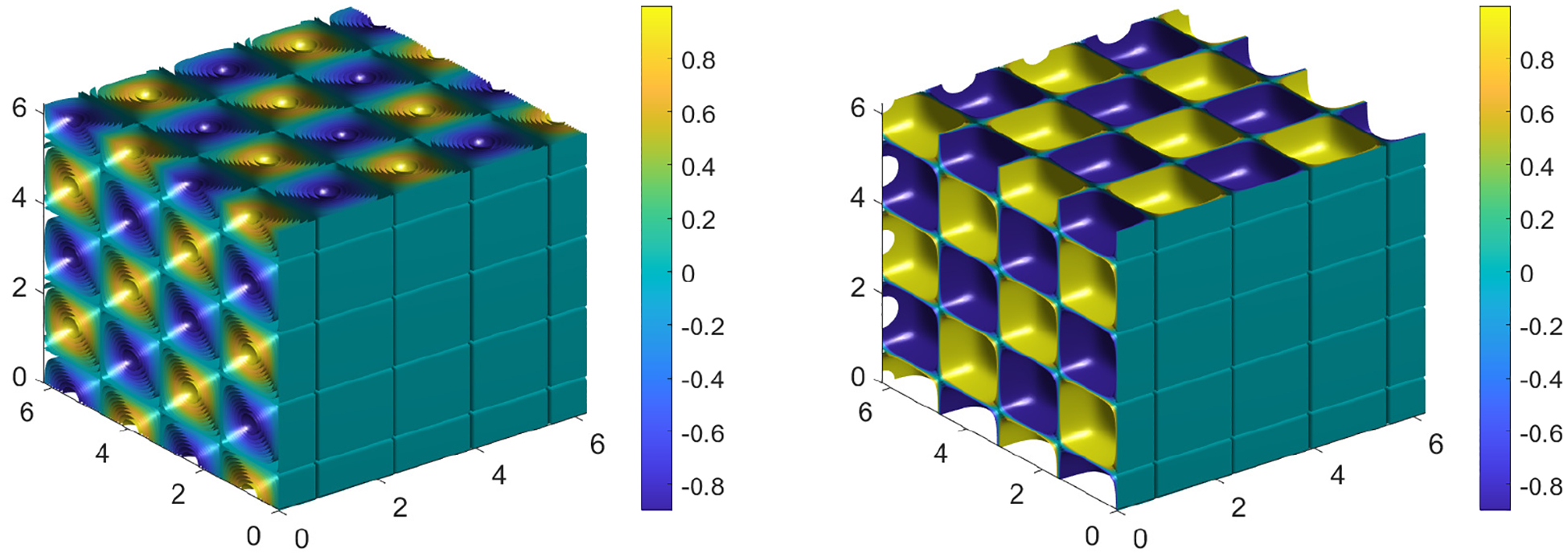
3D plots for u(x,y,z) and f(x,y,z) corresponding to Eq (5.1) with N=256.

We now extend the formulation for the Poisson problem in full 3D space variables. We solve the same problem given by Eq (5.1) on $\Omega =[0,2\pi {]}^{3}$ with the corresponding exact solution $u=\text{cos}\left(2x\right)\;\text{sin}\left(2y\right)\;\text{cos}(2z)$ and $f=-11.9\;\text{cos}\left(2x\right)\;\text{sin}\left(2y\right)\;\text{cos}(2z)$ as for simplicity, we have adopted similar form for u as we move from the 2D to 3D problem. Corresponding 3D plots for u and f are given in Figure 7. We solve the Poisson equation in 3D by using the spectral scheme given by Eq (5.5). To declare variables on the GPU, the simple MATLAB command can be used

U=cos(2*X).*sin(2*Y).*cos(2*Z);U=gpuArray(U);
F=−11.9*cos(2*X).*sin(2*Y).*cos(2*Z);F=gpuArray(F);

which then loads up the solution u and forcing term f into the GPU so that computations can be performed using the multiple cores provided by GPU architecture. For more details of the actual code used, please see the Appendix. Computation time for both CPU and GPU are presented in Table 3.

From Table 3, we learn that it is possible to speed up computations by a factor of about 2X using the GPU. We also note that for a single execution, CPU and GPU computations could be comparable; in fact, the CPU can be very competitive to the GPU in terms of computation time for the case of N=512. This is attributed to the fact that the GPU memory for the 2060 at N=512 is reaching its maximum capacity of about 8 GB and the CPU has 64 GB of on-board memory. The slowing down in computation at N=512 is not observed when using a more capable GPU with higher memory, as shown in the third column of Table 3. These computation times are for the A6000 (a higher-end GPU with 48 GB of memory). GPU computations allow to speed up computations by several factors, as shown in the recent work from Liu et al. related to CH problems [32]. Other GPU accelerations were reported in the work of Lam et al. for the thin-film TF equation from lubrication theory [33]. In the next section, we will show how this is possible by using the BHM method along with simple commands that will allow GPU computations.

### 5.3. Cahn-Hilliard equation 3D

**Figure 8.**
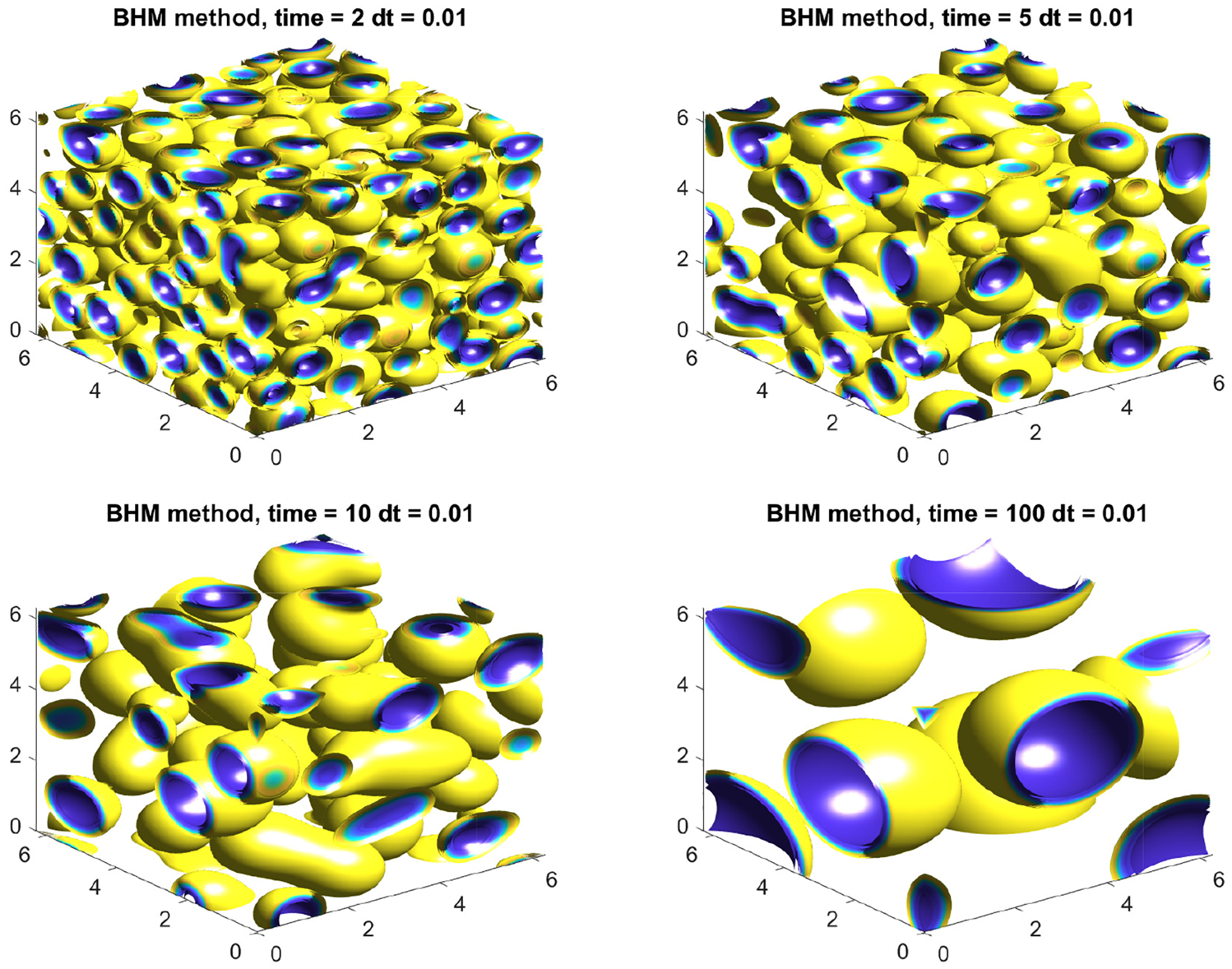
Numerical solution to the CH equation in 3D using BHM method with parameters N=256, ϵ=0.05, h=0.01M=1, $M_{1}=5$, $t_{f}=100$, and $\Omega =[0,2\pi {]}^{3}$.

We consider the CH equation in full 3D space variables using the BHM scheme (3.10) subject to periodic boundary conditions. We consider the CH equation (2.3) with an initial condition representing a mixed state of materials with u≈0.5 given by

(5.6) $$u\left(x,y,z,0\right)=0.5+0.01\text{rand}\left(x,y,z\right),$$

with ϵ=0.05, $M_{1}=5$ on $\Omega =[0,2\pi {]}^{3}$ using N=256. The simulation dynamics with a final computation time of $t_{f}=100$ are presented in Figure 8. The simulation illustrates the phase-separation process of a binary mixture in full 3D space dimensions with uneven volume fraction of materials. The circular phase, which is traditionally expected in 2D simulations, has translated into spheres in full 3D computations.

In addition to showcasing the schemes in 3D, we are also interested in speeding up the computations to counter act the more expensive computations associated with the larger arrays that result from the additional dimension. It is also relevant to explore ways that are efficient, yet easy to implement, for speeding up the computations for the case of large-scale dynamics or long-term behavior of solutions [33–36]. We concentrate on the computation presented in Figure 8 for $t_{f}=100$. This computation, with the given time step and 10,000 iterations, presents a good opportunity to benchmark the computational performance of the GPU implementation. Our reference point will be the computation time required by our CPU alone, which is an Intel-Core i9 with Windows 10 and 64 GB of ram. We will then use four Nvidia GPUs, two of which are consumer grade: the 2060 MaxQ and the 2070S, both with 8 GB of memory. The other two GPUs, the A6000 with 48 GB of memory and the A100 with 80 GB of memory, are enterprise. We solve the CH using the BHM method with a final time of $t_{f}=100$ using the CPU and the four GPUs and summarize our results in Table 4. The results show that it is possible to speed up 3D phase-field model computations by a factor of 8x to 15x if we simply consider the modest GPUs 2060 and 2070. We note that these speed ups are already substantial and that these GPUs are entry level with very easy access from a commercial point of view. On the other hand, if one has access to the more advanced enterprise GPUs like the A6000 and A100, then speeds ups ranging from 25x to 80x are certainly possible. We note that the last row in Table 4 contains approximated values obtained via extrapolation for CPU, 2060 and 2070S GPUs based on N=256. For those three devices, the computations took longer than one day due to hardware reaching maximum capacity (N=512).

### 5.4. Computed dynamics

We also include a simulation in which we solve the CH equation and consider even volume fractions with the use of the following initial condition

(5.7) $$u\left(x,y,z,0\right)=0.1+0.01\text{rand}\left(x,y,z\right).$$

**Figure 9.**
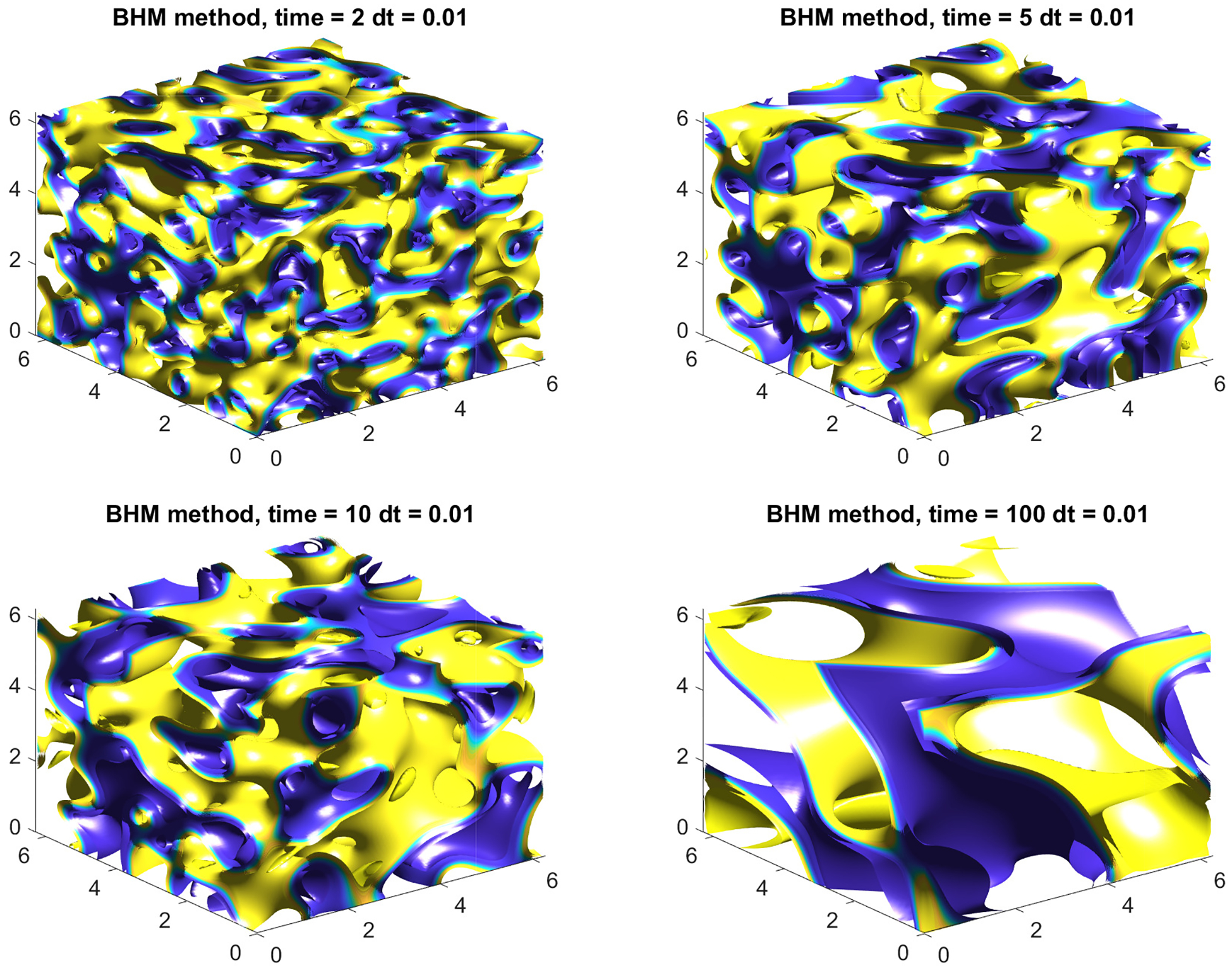
Numerical solution to the CH equation in 3D using BHM method with parameters N=256, ϵ=0.05, h=0.01M=1, $M_{1}=5$, $t_{f}=100$, $\Omega =[0,2\pi {]}^{3}$.

Using the same parameters as the previous 3D problem, we compute the numerical solution using the BHM method (3.10), and the results are presented in Figure 9.

**Figure 10.**
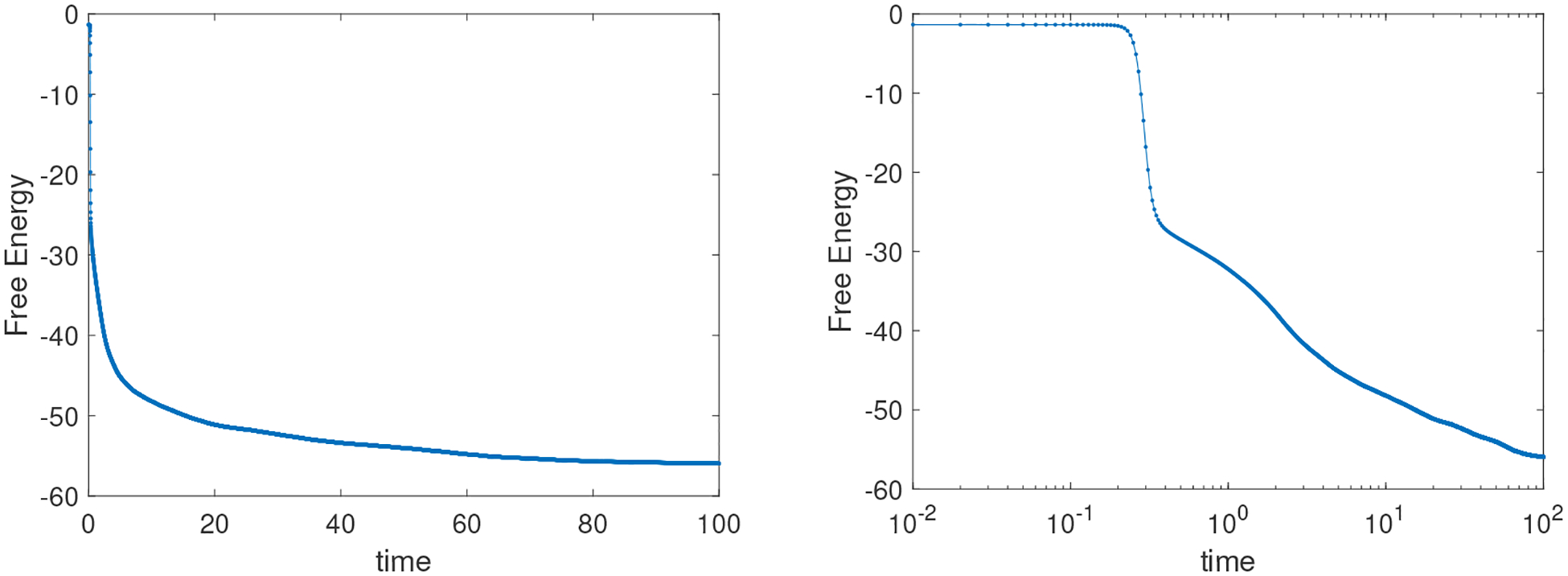
Energy evolution corresponding to simulation in Figure 9. Left and right figures are presented in regular and semilog scales to illustrate the energy-decreasing property.

It can be seen that the expected dynamics for a binary mixture with near equal volume fractions, u≈0.1, will undergo full separation without sphere formation. But, before reaching a steady-state, a meta-stable structure that resembles stripes in higher dimensions [11, 37] at around t=100 is obtained. The BHM method retains the energy-decreasing property during the entire simulation run, as shown in Figure 10.

**Figure 11.**
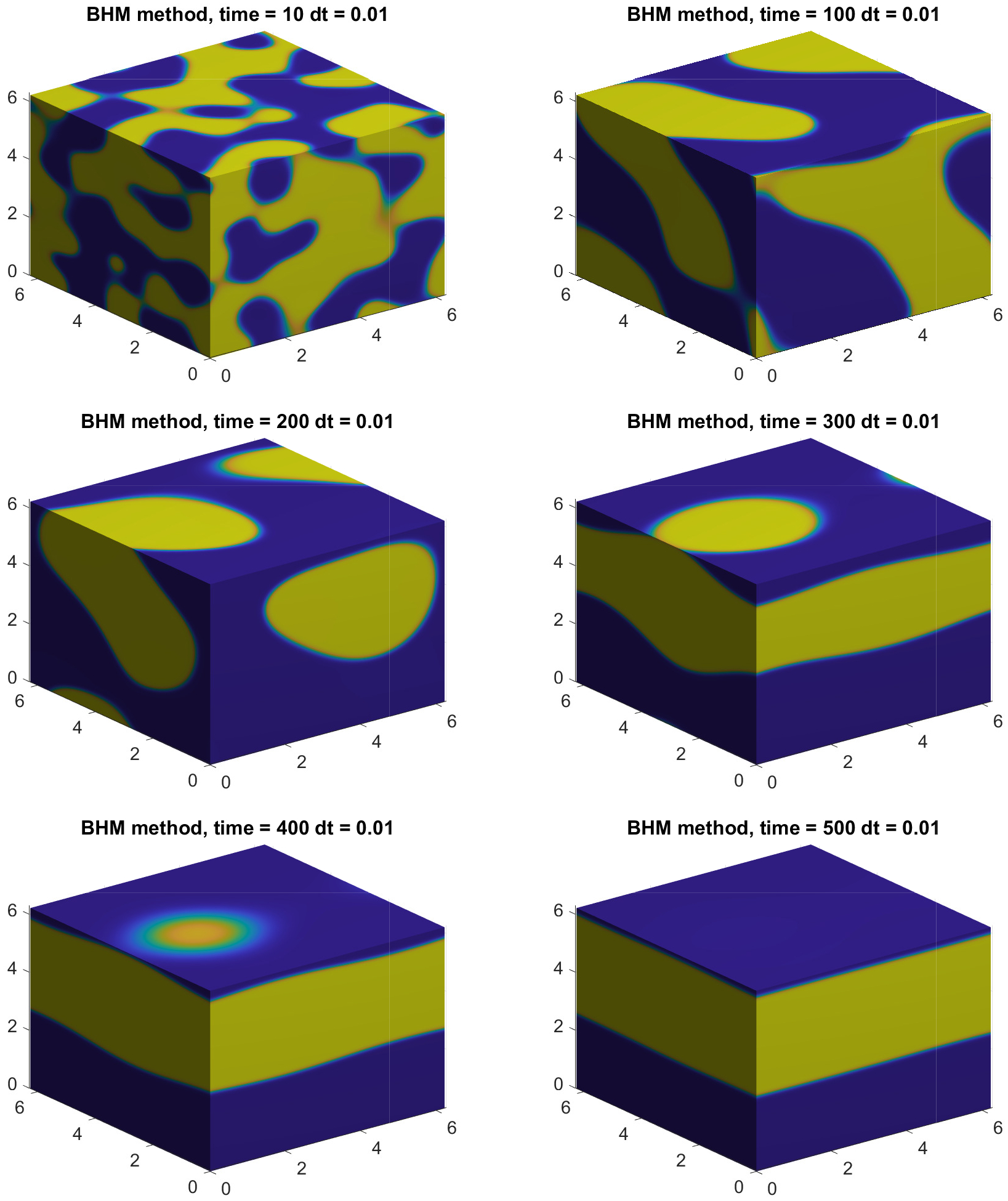
Extended numerical solution corresponding to Figure 9 using slices of the computational domain to capture the full separation process for the CH equation in 3D.

The dynamics of the solution to the CH equation should reach a full separation for long enough simulation runs, since it is modeling the separation of two materials [1]. In order to fully understand the evolution of the CH equation in 3D and to get the correct representation of the plots presented in Figure 9, we have added slices to those results and extended the simulations to better illustrate the separation process. Figure 11 represents the solution for t=10,100,200,300,400,500. Full separation of materials is observed at t=500, where slices have been placed at x=0,y=0 and z=2π. We note that for other phase-field models, full separation of materials may be prevented by material architecture and other modeling conditions [11–13].

**Figure 12.**
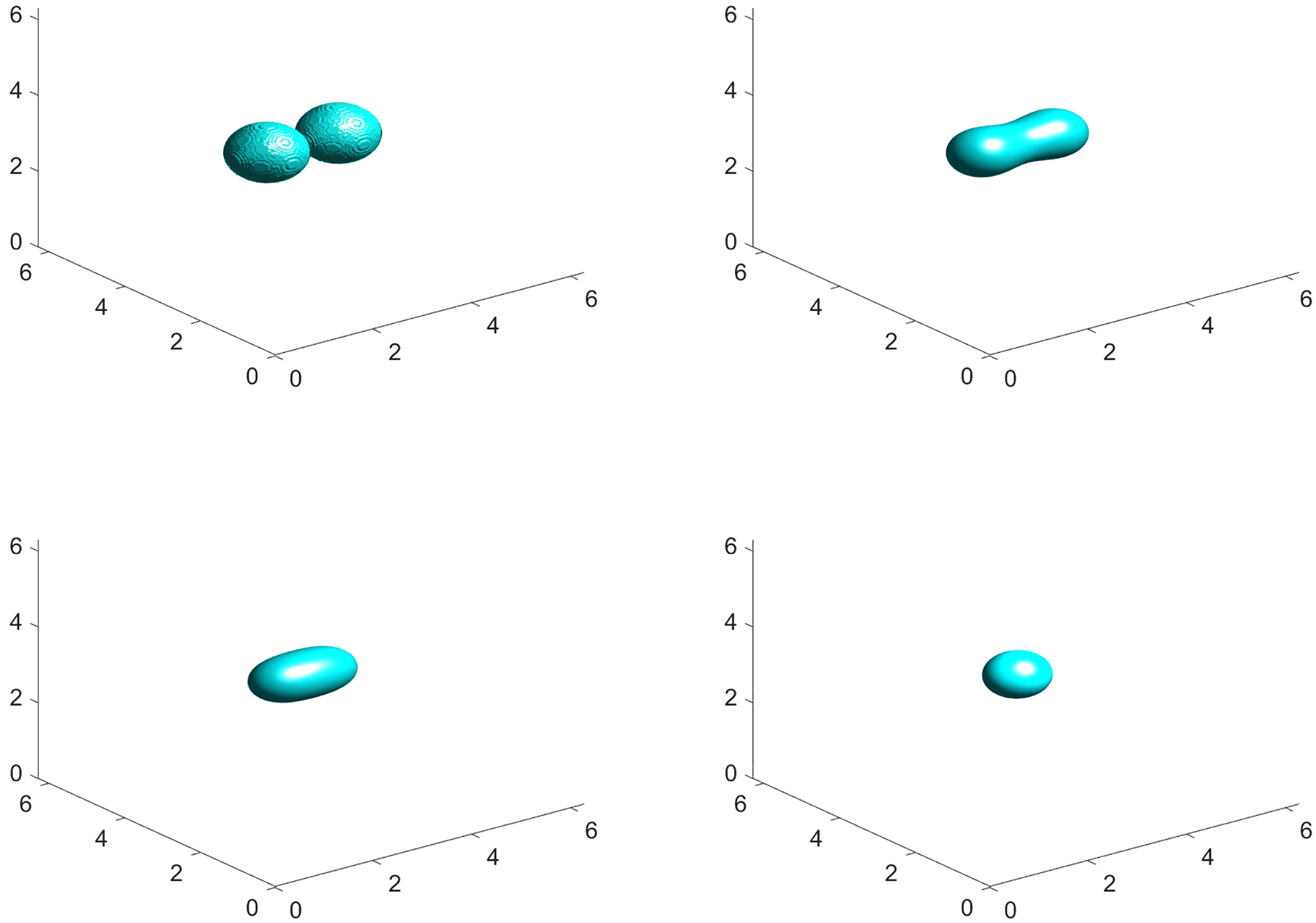
Numerical solution to the CH equation (2.3) in 3D illustrating the collision of two drops using the BHM-IMEX method with parameters ϵ=0.05, N=256, h=0.01M=1, $M_{1}=5$, $\Omega =[0,2\pi {]}^{3}$. Snapshots taken at t=0,2,5,10 (left to right and top to bottom).

For the final simulation example, we extend the drop coalescence in 3D space dimensions and test the performance of the proposed second-order method BHM-IMEX on $\Omega =[0,2\pi {]}^{3}$. Initial condition is given by

(5.8) $$u(x,y,t=0)=\left\{\begin{array}{ll} 1, & (L/9{)}^{2}<(x-L/2.8{)}^{2}+(y-L/2{)}^{2}+(z-L/2{)}^{2},\\ 1, & (L/9{)}^{2}<(x-b/1.7{)}^{2}+(y-L/2{)}^{2}+(z-L/2{)}^{2},\\ -1, & \text{otherwise,} \end{array}\right.$$

where L=2π. The simulation results are presented in Figure 12 with snapshots taken at t=0,2,5,10. An energy-stable solution is obtained while capturing the correct dynamics in full 3D space dimensions. Similar benchmark problems in 3D can be found in the work of Liu et al. [32].

### 5.5. GPU remark

We tested several strategies for accessing the GPU in the most efficient way. One option to perform a GPU computation and generate a plot was to save the end result of the GPU computation as a data file, then follow up with the CPU loading up that file, and generating a plot. The basic idea is summarized with the following sample code


save(‘U_numerical’,’U’);   %end of GPU computation
load(‘U_numerical’,’U’);   %CPU loading
isosurface(U);                                         %CPU plotting


### 5.6. CH equations with logarithmic potential

To further explore the main benefits of using the BHM methods as our main basis for the proposed approach to accelerate computations, we also include a more complicated case of the CH equation. That is, we consider the logarithmic case of the potential as done by Dai [38]. We consider the type of potential for the CH equation that has singularities at u=±1.

$$W_{\text{log}}\left(u\right)=\frac{\theta }{2}\left[\left(1+u\right)\text{ln}\left(1+u\right)+\left(1-u\right)\text{ln}\left(1-u\right)\right]+\frac{1}{2}\left(1-u^{2}\right),$$

where we choose θ=3/4 to attain inflection points at u=±1/2. The above potential has been proposed since the early developments of the CH equation, but this form of potential is not as popular as the polynomial type ($W(u)=1/4u^{4}-1/2u^{2}$). This potential has a local maximum on u=0 and minimum values approaching u=±1. Hence, mixtures near the spinodal region will evolve into the pure phases but, due to the singular nature of the potential, overshoots or undershoots are prevented on [−1, 1]. Hence, for this type of potential, one expects for the solution u to be in-fact bounded on [−1, 1]. We tested the adaptability of our schemes by solving the Cahn-Hilliard equation with a singular potential. The nonlinear contribution to the CH equation with logarithmic potential becomes

$$W_{\text{log}}^{\prime }\left(u\right)=\varphi \left(u\right)=\frac{3}{8}\text{ln}\left(\frac{1+u}{1-u}\right)-u.$$

Applying the BHM formulation to the CH equation with logarithmic potential then gives the following scheme

(5.9) $$\hat{U_{n+1}}=\frac{\hat{U_{n}}-h\epsilon^{2}\left(M(u)-M_{1}\right)k^{2}\hat{U_{n}}-M(u)k^{2}\left[\hat{\varphi \left(U_{n}\right)}\right]}{1+hM_{1}\epsilon^{2}k^{4}}.$$

**Figure 13.**
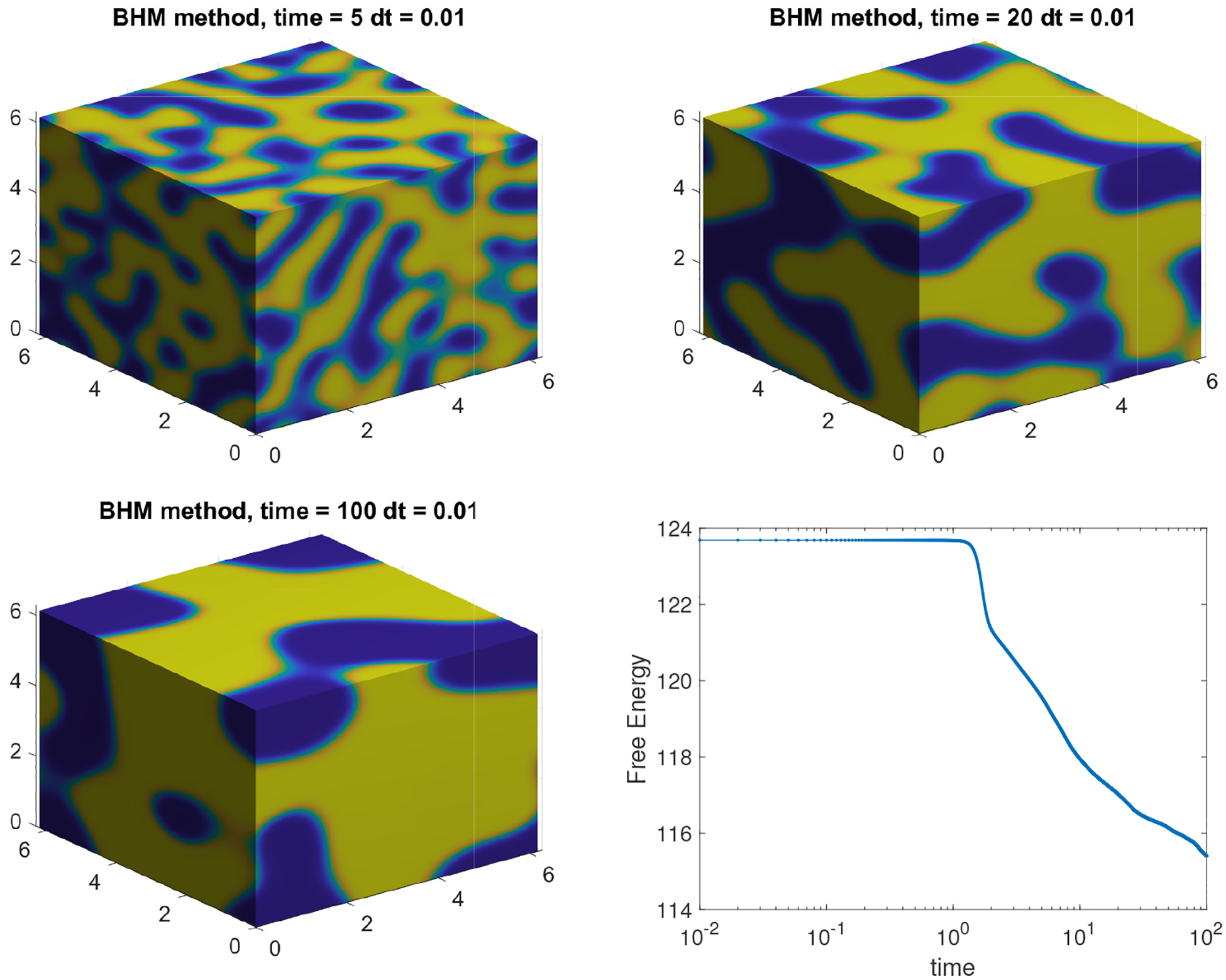
Numerical solution to CH equation with logarithmic potential. Parameters used $\Omega =[0,2\pi {]}^{3}$ with N=256, M=1, $M_{1}=5$ and ϵ=0.05.

We consider the CH equation with logarithmic potential on $\Omega =[0,2\pi {]}^{3}$ with N=256, M=1, $M_{1}=5$, and ϵ=0.05 using the initial condition from (5.7). Simulation snapshots are presented in Figure 13. The simulation undergoes phase separation in full 3D and this is done while preserving a bound on the solution u on [−1,1] due to the structure of the logarithmic potential. Solution boundedness under singular potentials is beyond the scope of this paper, but we can refer the readers to studies using a free boundary formulation for the CH equation under the case of different mobilities and different potentials [34, 38].

## 6. Concluding remarks

We presented numerical approaches for phase-field models that are efficient, accurate, and easy to implement. The methods retain the energy-decreasing property and provide small errors. The BHM method is very efficient and shares the ease of implementation as the CS method. A second-order method based on the BHM was introduced using an IMEX formulation. The methods were tested with a variety of benchmark problems in 2D and 3D for short, and long-term simulations demonstrating their good performance. In addition to the classic CH equation with polynomial potential, we also demonstrated how the BHM easily applies to the case of a logarithmic potential. One of the main advantages for the BHM method is that the nonlinearities associated with a more complicated potential are easily handled at the current time level (explicitly). This is a drawback for the CS method, since separating the energy into convex and concave parts may be not possible. With our proposed work, users can choose a lower-accuracy method with the choice of a smaller h if accuracy requirements are modest. On the other hand, users can choose the higher-order schemes (with slightly higher computation cost) if accuracy demands are higher. We note that all schemes presented here are very efficient and can run in a modest laptop. The presented methods can be used as a powerful tool to study a large class of phase-field models. For speeding up the computations, we found that with modest GPU equipment, 8x and 15x speed ups are possible with the use of a few simple commands in MATLAB to access the GPU. Using higher-end equipment, it is possible to reach 25x or even 80x speed ups. We believe that the CPU and GPU implementation examples, with actual code that the users can use openly, could serve as a powerful tool for a broad range of researchers interested in efficient computational aspects of phase-field models. While the main phase-field model studied in this paper was the CH equation, a large class of phase field-models, including the BCP equation, phase field crystal (PFC) equation, the functionalized Cahn-Hilliard (FCH) equation, and the thin-film (TF) equation [11, 13, 39–43], can be approximated with the BHM approach. Many useful studies could benefit from the work presented here; in particular, large-scale computation of complex phase-field models and complicated simulations in full 3D space dimensions. Future work will include the rigorous analysis for the schemes with regards to energy-decreasing property and convergence analysis of the scheme, as well as the applicability of the methods to GPU accelerations in systems of CH equations [44] and systems of aggregation equations [45].

## Figures and Tables

**Table 1.** CPU Intel-Core i9 computation time in seconds for the different cases of N for solving the CH equation problem simulating drop collision using h=0.01 and $t_{f}=50$.

N	CS	BHM	IMEX
256	13.76	13.62	18.51
512	18.18	23.94	36.45
1024	59.17	120.74	149.13

**Table 2.** CPU Intel-Core i9 computation time in seconds for the different cases of N using the pseudo-spectral approach for solving the Poisson problem in 2D.

N	CPU
128	0.0019
256	0.0032
1024	0.026
2048	0.11
4096	0.43
8192	1.91

**Table 3.** CPU Intel-Core i9 and GPU computation time in seconds for the different cases of N using the pseudo-spectral approach for solving the Poisson problem in 3D.

N	CPU	2060 Max-Q	A6000
32	0.0018	0.0016	0.0007
64	0.0051	0.0021	0.0017
128	0.037	0.0024	0.0026
256	0.35	0.0069	0.0058
512	2.79	2.47	0.089

**Table 4.** CPU Intel-Core i9 and GPU computation time in minutes for the different cases of N using the BHM approach for solving the CH equation in 3D with random initial condition.

N	CPU	2060 Max-Q	2070S	A6000	A100
64	6.27	0.81	0.63	0.45	0.17
128	56.67	6.68	4.45	2.26	0.72
256	444.42	52.84	34.74	17.03	5.33
512	3517.21	417.82	277.85	123.39	41.86

## Appendix

The following MATLAB codes are used to simulate the Poisson problem in 3D and the phase-separation process with random initial state for CH equation in 2D and 3D using the BHM scheme. After minor modifications, the codes can be easily extended for other simulations in this paper. The codes used for these experiments are available on the freely accessible GitHub repository https://github.com/sauloorizaga.

Detecting GPU

%To get info on GPU installed
gpuDevice

Poisson equation in 3D

%This code solves the Poisson problem in 3D with GPU
M=2;a=0;b=M*pi;N=128;h=(b-a)/N;n=N;
%xgrid formation (a b] and eventually (a b]^2
x=(a:h:b-h);[X,y,Z] = meshgrid(x,x,x);
 k=[[0:N/2] [-N/2+1:−1]]./((M)/2);
[k1x k1y,k1z]=meshgrid(k.^1,k.^1,k.^1);
[kx ky kz]=meshgrid(k.^2,k.^2,k.^2); k2=kx+ky+kz;k4=k2.^2;
 U=cos(2*X).*sin(2*Y).*cos(2*Z); U=gpuArray(U);%GPU
 F=−11.9*cos(2*X).*sin(2*Y).*cos(2*Z);F=gpuArray(F);%GPU
    uhat=(fftn(F))./(−1.*k2+.1);Usol=real(ifftn(uhat));
figure(1); isosurface(X,Y,Z,U); isosurface(X,Y,Z,U,.5);
isosurface(X,Y,Z,U,−.5); ax = gca; ax.FontSize = 14; figure(2);
isosurface (X,Y,Z,F); isosurface(X,Y,Z,F,.5);
isosurface(X,Y,Z,F,−.5); ax = gca;ax.FontSize = 14;

CH 2D with GPU

%This code solves the CH equation in 2D using the BHM method
dt=0.01;M1=2; iter=1; tfinal=5; N=256; a=0; L=2; b=L*pi;
%number of grid points N uniform mesh thickness
h=(b-a)/N; n=N;
%xgrid formation (a b] and eventually (a b]^2
x=(a:h:b-h);x=gpuArray(x);[X,Y]=meshgrid(x,x);
k=[[0:N/2] [-N/2+1:−1]]./(L)/2);k=gpuArray(k);
[k1x k1y]=meshgrid(k.^1,k.^1); [kx ky]=meshgrid(k.^2,k.^2);
k2=kx+ky; k4=k2.^2;
%Initial Condition----------------------
U=0.01*rand(N,N)+.5*0;U=gpuArray(U);
figure(1); pcolor(X,Y,U),shading interp,axis(‘off’),axis(‘equal’);
%parameters
epsilon=.05; eps2=epsilon^2;lhs=1+dt*M1*k4*eps2; % CH lhs
hat_U=fft2(U); it=0; j=0; nn=0; t=0.0; M=1;
while (t < tfinal) U1=U;
  RHS=eps2*(M1-M)*ifft2(k4.*fft2(U1))+ifft2(−1*k2.*fft2(U1.^3-U1));
  hat_rhs=hat_U + dt.*fft2(RHS);
  hat_U1=hat_rhs./lhs; U1=real(ifft2(hat_U1));
U=real(U1); hat_U=hat_U1; it=it+1; t=t+dt; %update
end %main loop
figure(2);ax = gca; ax.FontSize = 14;
pcolor(X,Y,U),shading interp,axis(‘off’),axis(‘equal’);
title([‘BHM method = ‘ num2str(t),’ dt = ‘ num2str(dt)], …
‘FontSize’,12);

CH 3D with GPU

%This code solves the CH equation in 3D using the BHM method
dt=0.01; M1=2; iter=1; tfinal=5; N=256;
a=0; L=2; b=L*pi;
%number of grid points N uniform mesh thickness
h=(b-a)/N; n=N;
%xgrid formation (a b] and eventually (a b]ˆ2
x=(a:h:b-h); x=gpuArray(x); [X,Y,Z]=meshgrid(x,x,x);
k=[[0:N/2] [-N/2+1: −1]]./((L)/2);k=gpuArray(k);
[k1x k1y k1z]=meshgrid(k.ˆ1,k.ˆ1,k.ˆ1);
[kx ky kz]=meshgrid(k.ˆ2,k.ˆ2,k.ˆ2);
k2=kx+ky+kz; k4=k2 .ˆ2;
%Initial Condition----------------------
U=0.01*rand(N,N,N)+.5*0; U=gpuArray(U);
%plots IC
figure(1); isosurface(X,Y,Z,U); isosurface(X,Y,Z,U,−.5);
isosurface(X,Y,Z,U,.5)
%parameters
epsilon=.05; eps2=epsilonˆ2;
lhs =1+dt*M1*k4*eps2; % CH lhs
hat_U=fftn(U); it=0; j=0; nn=0; t=0.0;
M=1;
while (t < tfinal)
U1 = U;
  RHS=eps2*(M1-M)*ifftn(k4.*fftn(U1))+ifftn(−1*k2.*fftn(U1.ˆ3-U1));
  hat_rhs=hat_U + dt.*fftn(RHS);
  hat_U1=hat_rhs./lhs;
  U1=real(ifftn(hat_U1));
U=real(U1); hat_U=hat_U1; it=it+1; t=t+dt; %update
end %main loop
figure(2); isosurface(X,Y,Z,U); isosurface(X,Y,Z,U,−.5);
isosurface(X,Y,Z,U,.5);ax=gca;ax.FontSize=14;

**Remark:** The presented codes with use of GPU can run in non-GPU devices by simply commenting out the associated GPU declaration commands X=gpuArray(X), where X is the array in question. We also note that the codes have been given in the Appendix for maximum reproducibility of the results presented in this paper. The BHM approach can be easily adapted to more complicated phase-field models (BCP, PFC, and TF equations) as explained in the main body of the manuscript. For users running extensive simulations, we recommend the GPU strategy presented in Section 5.5. The authors welcome conversations or questions about accelerated computations for phase-field models.
